# By the Way

**Published:** 1911-04-22

**Authors:** 


### By the Way.

### AS OTHERS SEE US.

The last word in comfort in the organisation of the home will always remain with the English. No one will doubt this who has read the communication in which our collaborator, Dr. G. West-Hecq, of Namus, has informed us: "An English society has just taken out a patent for the installation of crematorial furnaces in the home. An English house will no longer be complete unless it possesses, in addition to its bathroom, its funereal 'grill-room.' The circular published by the new company describes the advantages of its installation in moving and almost engaging terms. It resembles 'a gas-cooker, low but long, covered by a steel case into which the coffin is introduced.' The cremation itself is only a question of a few seconds, but the furnace must be heated an hour beforehand. To those who do not as yet possess at home this 'last word' in modern comfort, the company offers to hire its apparatus." Here in truth is an idea which could only have arisen in the brain of an Anglo-Saxon. Business is business!—*La Chronique Médicale.*

[Someone, in vulgar parlance, has apparently been pulling our contemporary's leg! If not, what is the name of this enterprising firm, where are its offices and what are the names of its directors and promoters? We sometimes hear a good deal about ourselves from the foreign Press. Only recently a Continental medical paper announced "the establishment at a cost of £100,000 of a magnificent new surgical home in London!"]

### FOOD ADULTERATORS IN 1481.

"Poor people who frequent fairs and markets are often deceived and swindled by country folk who sell tainted or adulterated commodities, such as rotten and addled eggs, skimmed and watered milk, butter containing turnips and stones. . . ." Such is not, as might be supposed, the complaint of a twentieth-century housewife, but the actual text of an appeal which the consuls, burghers, and townsfolk of Ambert made in 1481 to "Messire Jacques de Tourzel, seigneur d'Allegre, de Viverols, de Riols et du pays de Livradois, de Saint-Just, de Chomelys et autres terres." The four consuls explained at length to their lord the trickery and thefts which were being perpetrated in their town. "Complaints are always being made, and law suits and quarrels take place unceasingly. Tumults, disputes, and discussions are rife in the town; because of, and on the occurrence of, the deeds mentioned above, burghers and countryfolk wrangle and dispute together." They bring their petition to a close by imploring him to put an end by energetic measures to a situation so intolerable. Jacques de Tourzel has no hesitation in complying. Heartily wishing "to be quit of such ugly and abominable practices and to punish severely such delinquent people," he "wishes and ordains" that the following punishments should be inflicted in the three cases enumerated below:—

"In the case of every man or woman who has sold watered milk, a funnel shall be placed in his throat and the aforesaid milk poured down it, until such time as a physician or barber-surgeon shall state that he is unable to swallow more without danger to life.

"Every man or woman who has sold butter containing turnips, stones, or anything of a like nature, shall be seized and very carefully attached to our pillory of Pontel. The aforesaid butter shall then be roughly placed on his head and there left so long as the sun has not completely melted it. Dogs shall be allowed to come and lick it, and vulgar people permitted to insult him with such defamatory epithets as shall please them best (without offence to God, the King, and others). If the weather be unfavourable and the sun not sufficiently warm, the aforesaid delinquent shall be exposed in the large hall of the gaol before a large fire, in such a manner that all may see.

"Each man or woman who has sold rotten or addled eggs, shall be seized and taken to our pillory of Pontel. The aforesaid eggs shall then be handed over to little children, who, by way of a joyful pastime, shall vie with each other in throwing them at his face and clothes, in such a manner as to make everyone laugh. But that they shall not be allowed to throw any other form of filth."—*Journal des Débats.*

### THE ORIGIN OF RACES OF COLOUR.

"God put water to boil in a great cauldron. In this he dropped one of those large cockroaches called *drapo-drapo.* He took it out almost at once, but under the action of the boiling the black and shining shell of the animal became detached from its body. God said 'That is just right.' He then gave the signal to three men of indeterminate colour who had been holding themselves in readiness, immobile, and drawn up in a line a little distance away. 'Run and jump in there,' said God to them pointing to the bubbling liquid. The first did not hesitate, but with head down threw himself boldly into the copper. He emerged parboiled, skinned, but whitened. He became a white man, a man of superiority. Emboldened by his example, the second drew near, but without haste and without great enthusiasm, carried out his plunge into the fatal bath. He emerged red, empurpled from head to foot by the blood of his predecessor. He became a red-skin. The third, however, was frightened at the test, and could not gather up sufficient courage to make the necessary plunge. God was obliged to scold him. All this took time, and the water was evaporated, reduced, and concentrated. It became thick, dirty, and black, so that the last sufferer withdrew from his too long delayed bath less clean than he entered it. His skin became dark, dirty, and black. He was the first of the negroes." Thus spoke Pachiola, Tamouchi of the Emerillons, natives of French Guiana, when confiding this quaint legend to Dr. Tripot (Paris, Plon-Nourrit, 1910).—*L'Ami du Médecin.*

